# Sebaceous adenoma of the eyelid: A clinical, histopathological, and immunohistochemical perspective

**DOI:** 10.18632/oncoscience.655

**Published:** 2026-04-02

**Authors:** Gunvanti Rathod, Monica Mishra, Banka Sai Swetha, Siddharam S. Janti, Shamli D. Zalke, Kolavali Raghavendra Rao, Alisha Khan

**Affiliations:** ^1^Department of Pathology and Lab Medicine, All India Institute of Medical Sciences (AIIMS), Bibinagar, Hyderabad 508126, India; ^2^Department of Ophthalmology, All India Institute of Medical Sciences (AIIMS), Bibinagar, Hyderabad 508126, India

**Keywords:** sebaceous adenoma, eyelid, histopathology, Ki-67, EMA

## Abstract

Sebaceous adenoma (SA) is a rare, slow-growing benign tumor arising from sebaceous glands, accounting for less than 0.5% of all cutaneous neoplasms and approximately 1–2% of eyelid tumors. The eyelid is an uncommon site for this lesion. Histologically, SA shows well-circumscribed lobules composed of mature sebocytes with vacuolated cytoplasm and a peripheral rim of basaloid cells, without nuclear atypia or mitotic activity. We report a case of an 81-year-old man with a unilateral papillomatous lesion in the left lower eyelid, diagnosed as sebaceous adenoma on histopathology. Immunohistochemistry revealed EMA positivity and a low Ki-67 proliferative index, confirming its benign nature. The rarity of its pseudopapillomatous presentation and its need for differentiation from sebaceous carcinoma highlight the diagnostic importance of this case.

## INTRODUCTION

Sebaceous adenoma (SA) is an uncommon benign adnexal tumor derived from sebaceous glands, most frequently located on the face, scalp, and trunk. The eyelid represents one of the rarest periocular sites due to the abundance of Meibomian and Zeiss glands. Among the periocular tissues eyelid is one of the most frequent locations. Other such common locations are face or scalp [1]. SA is typically asymptomatic but can present as a slow-growing, painless mass, often resembling tumor of epidermal or stromal origin and sometimes metastatic [2]. Physical examination reveals yellowish, smooth, well-circumscribed soft or firm nodules on the eyelid margin measuring a size of few millimetres to several centimetres in diameter [3]. SA is composed of both sebaceous (which contain lipid-rich cytoplasm) and basaloid cells (with dark-staining nuclei) which may have areas of cystic change, focal necrosis and calcification. Standard method of diagnosis is clinical examination and histopathology for confirmation. Immunohistochemical staining is typically positive for Adipophilin, AR, CK7 [4]. The distinction between sebaceous adenomas, sebaceoma and other tumors like sebaceous carcinoma is important for final diagnosis [5]. Although histopathologic features of SA are well recognized, variations in clinical morphology and age of presentation make each case diagnostically significant. This case emphasizes a pseudopapillomatous sebaceous adenoma of the eyelid in an elderly male, a presentation rarely documented in literature, and discusses its differentiation from sebaceous carcinoma and syndromic associations.

## CASE REPORT

**Figure 1:**
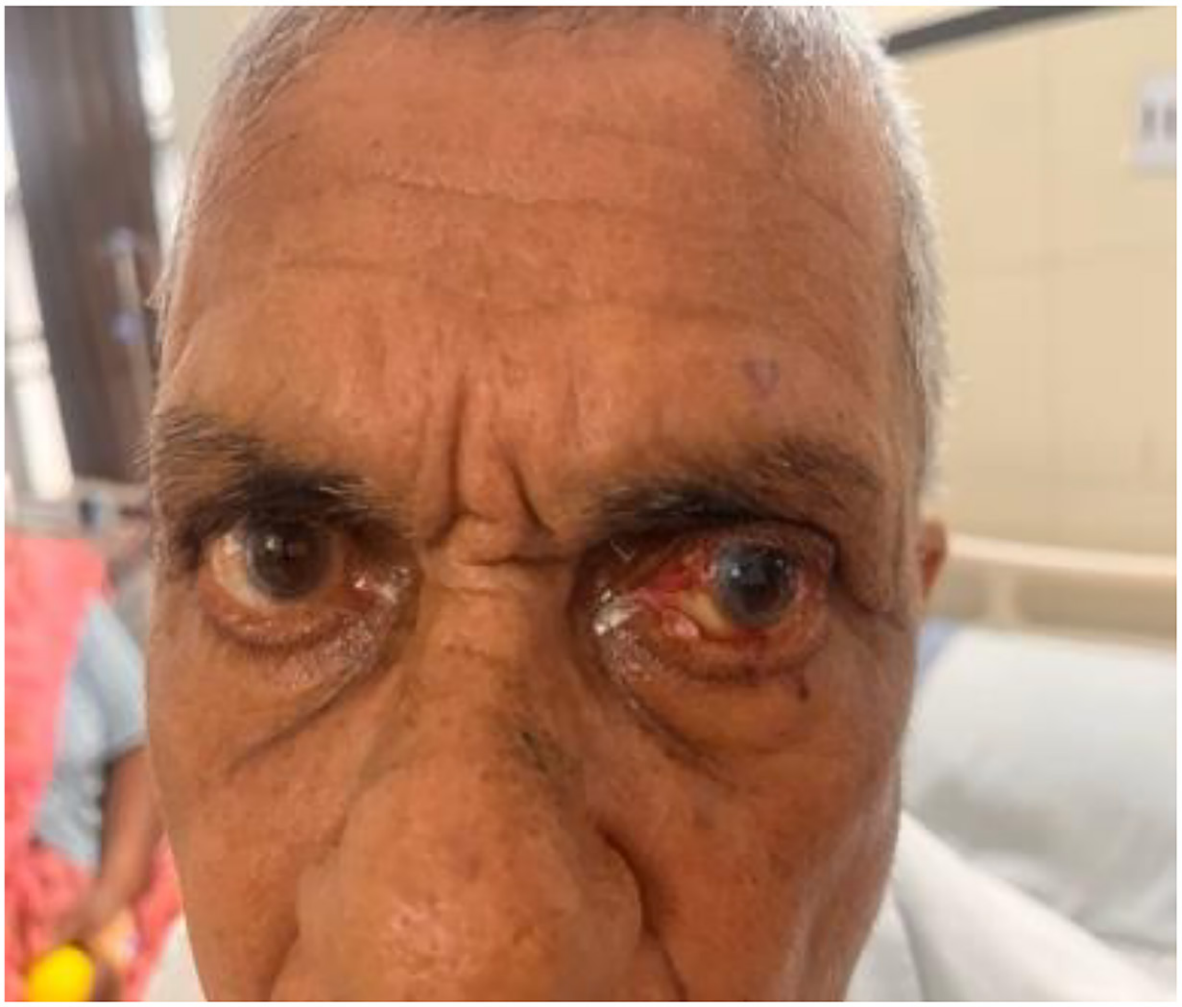
Clinical image showing yellowish papillomatous exophytic lesion in the left lower eyelid.

**Figure 2:**
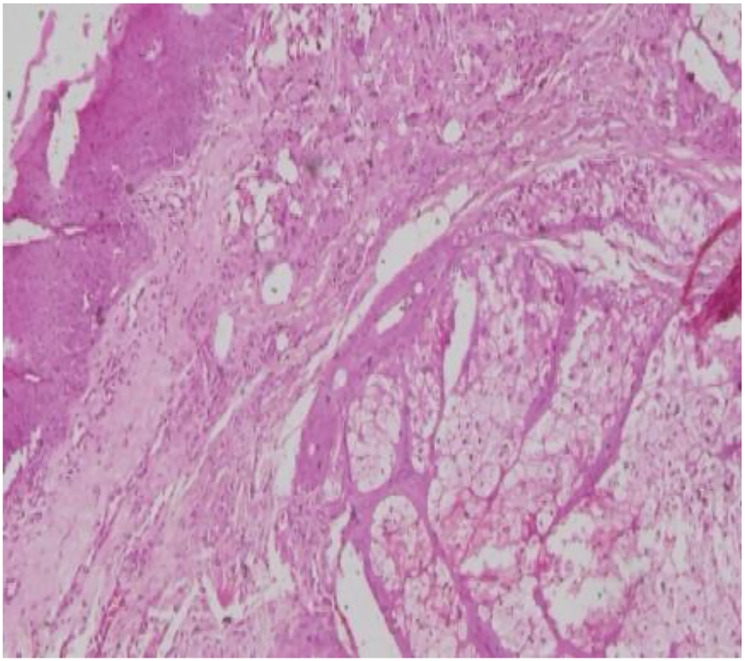
Low-power photomicrograph shows a well-circumscribed, lobulated dermal tumor composed of variably sized sebaceous lobules. The lobules demonstrate peripheral basaloid germinative cells with central areas of mature sebocytes showing abundant foamy/vacuolated cytoplasm. No significant cytologic atypia or infiltrative growth is identified (H&E, 10X).

**Figure 3:**
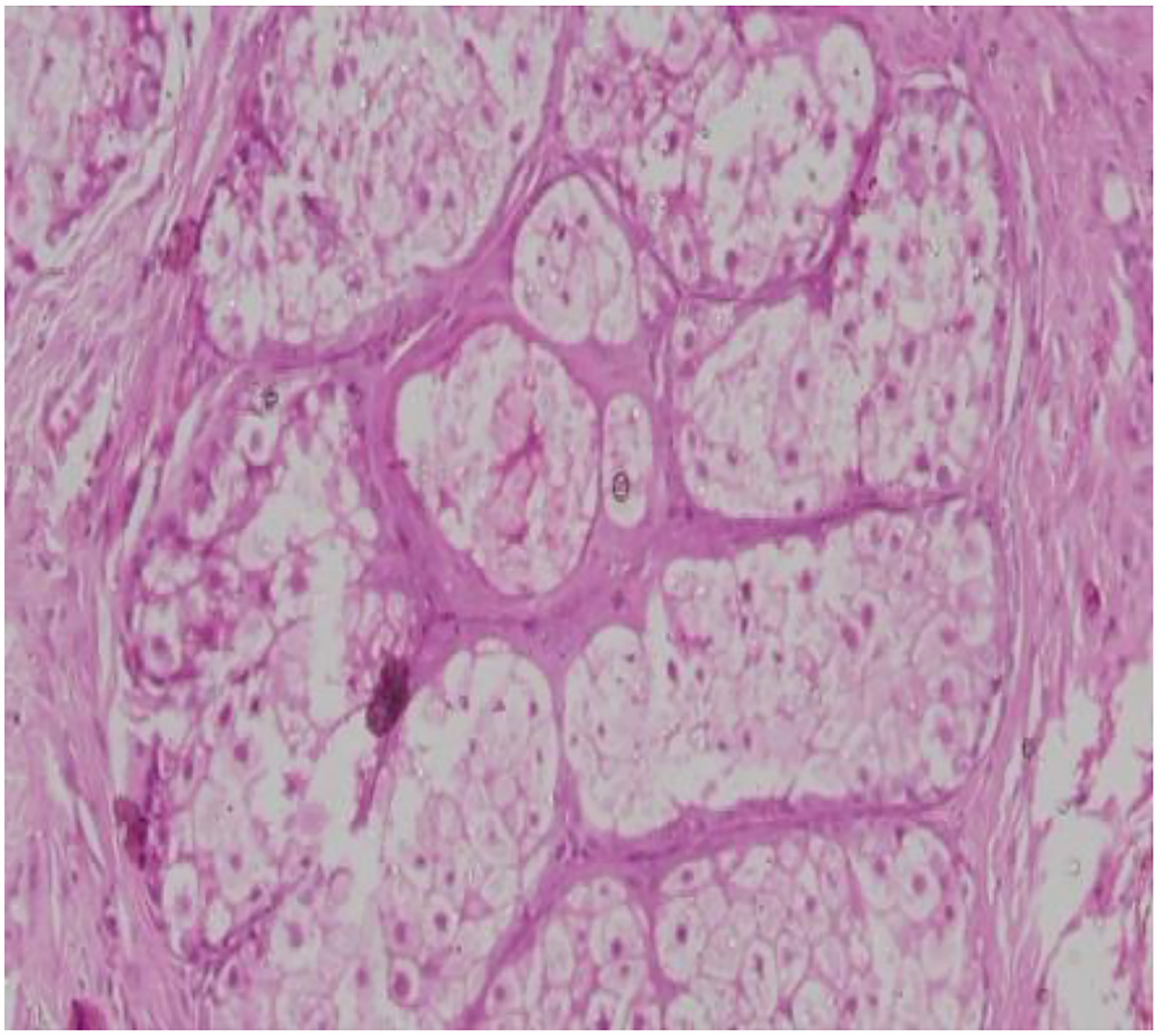
Section shows lobules of sebaceous gland proliferation with benign features [H&E, 10x].

**Figure 4:**
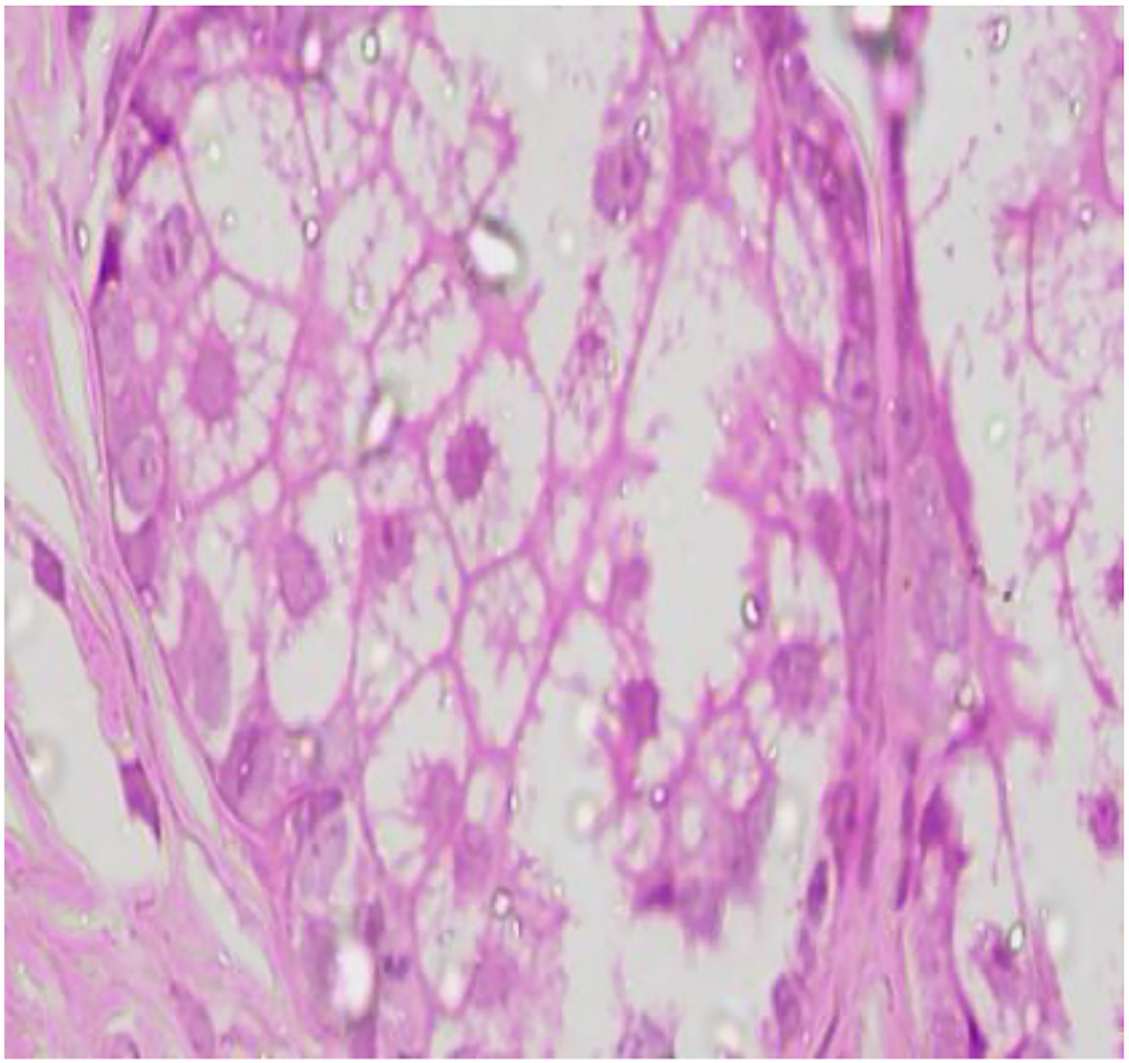
High-power photomicrograph shows sebaceous lobules composed of peripheral layers of small basaloid germinative cells transitioning toward centrally located mature sebocytes with abundant clear, multivacuolated (lipid-rich) cytoplasm and small, centrally placed nuclei. No significant nuclear atypia, necrosis, or increased mitotic activity is identified (H&E, 40X).

**Figure 5:**
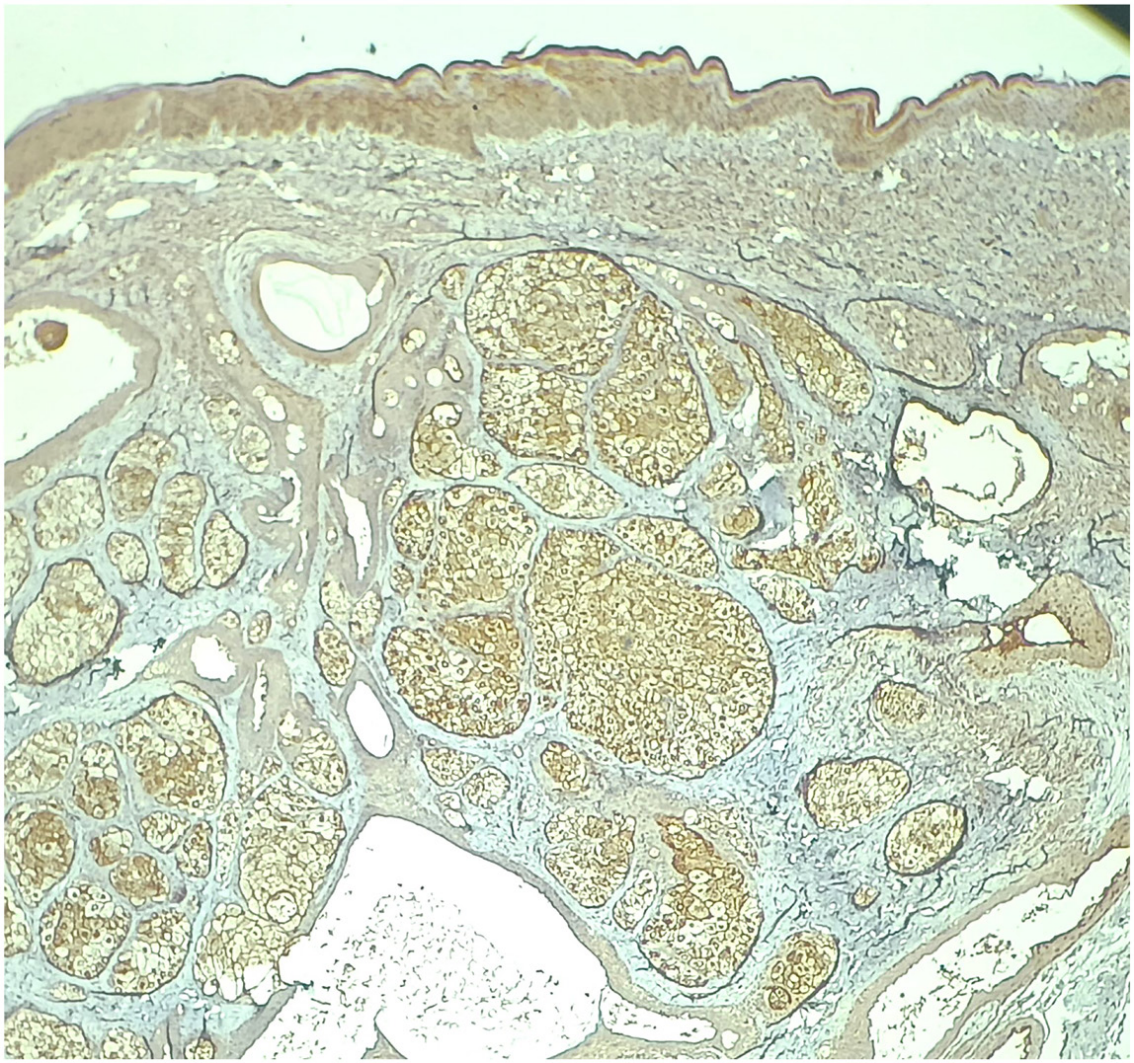
Immunohistochemistry for epithelial membrane antigen (EMA) demonstrates cytoplasmic positivity in mature sebocytes within the tumor lobules, highlighting sebaceous differentiation. (IHC, 4X).

**Figure 6:**
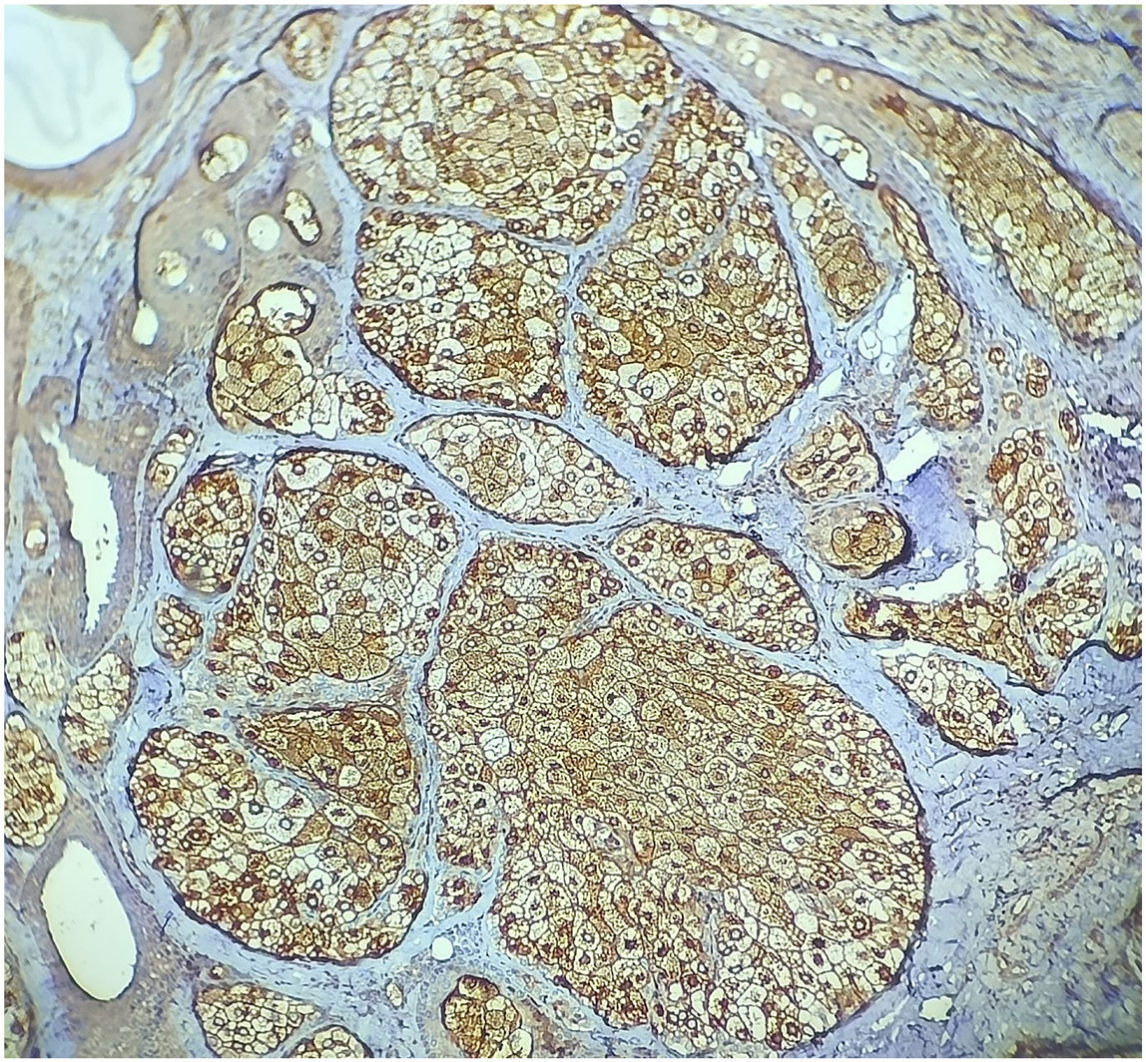
High power view of immunohistochemistry EMA positivity supports diagnosis of sebaceous adenoma. (IHC, 10X).

**Figure 7:**
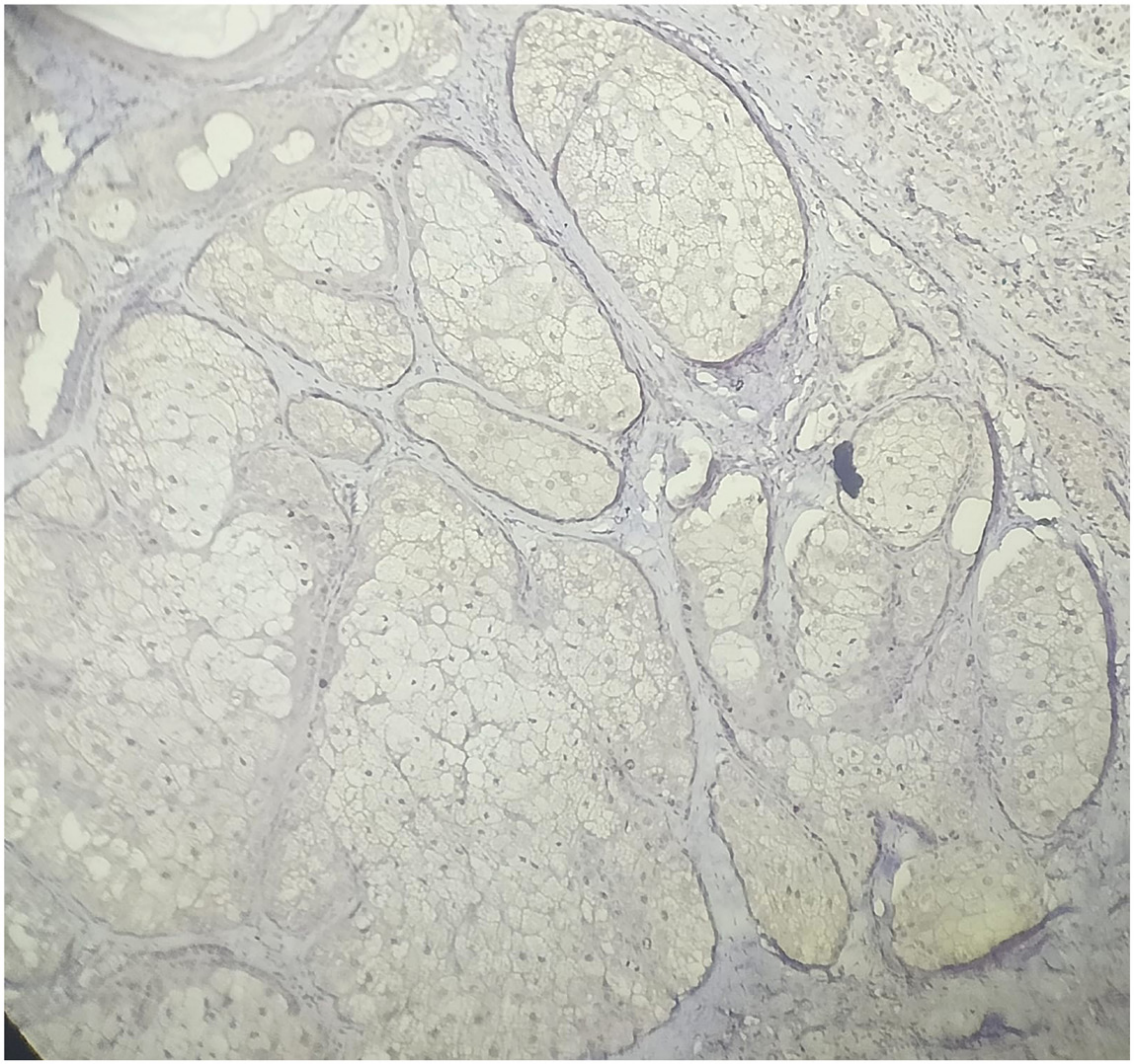
Low power view of Ki-67 immunohistochemistry shows mature sebocytes are largely negative. The low Ki-67 labeling index supports the benign nature of Sebaceous Adenoma. (IHC, 10X).

**Figure 8:**
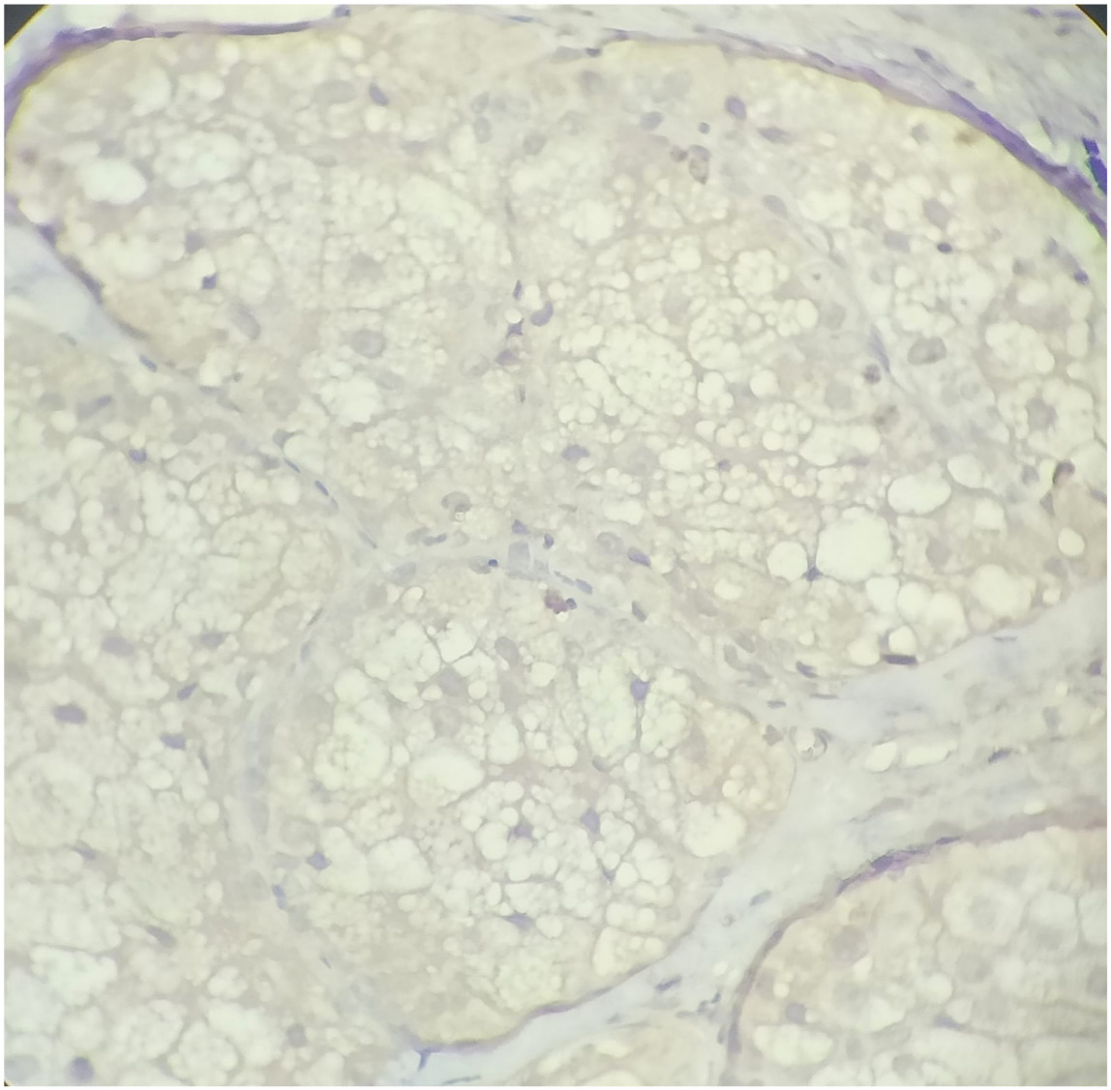
High power view of Ki-67 immunohistochemistry shows low proliferative activity (IHC, 40X).

An 81-year-old man presented to the ophthalmology outpatient department with complaints of blurring of vision in the left eye. Best corrected visual acuity was 20/40 in the right eye and 20/120 in the left. On examination, a 1 × 1 cm exophytic, yellowish-pink papillomatous lesion was noted on the left lower eyelid. The lesion had been slowly increasing in size for several years without pain or discharge. There was no prior history of similar lesions on the face or elsewhere and no personal or family history of visceral malignancy. The lesion was excised and submitted for histopathological and immunohistochemical analysis. Gross Findings showed a grey-white tissue fragment measuring 0.5 × 0.2 × 0.2 cm. Microscopic Findings showed the tumor was lined by stratified squamous epithelium. Subepithelial tissue showed well-circumscribed lobules of sebocytes with vacuolated cytoplasm and a peripheral layer of basaloid cells. No nuclear atypia, necrosis, or mitotic activity was noted (Figures 1–4). Immunohistochemistry for Epithelial Membrane Antigen (EMA) showed Diffuse cytoplasmic positivity confirming sebaceous differentiation and Ki-67 showed low proliferative index (<5%), supporting benign behaviour (Figure 5–8).

Based on morphology and IHC profile, the lesion was diagnosed as sebaceous adenoma. Screening for mismatch repair (MMR) gene deficiency (MLH1, MSH2, MSH6, PMS2) was discussed but deferred as the patient had no relevant family history or other lesions.

## DISCUSSION

Sebaceous adenoma is a benign neoplasm composed of lobules of sebaceous cells showing varying degrees of differentiation. Though histological features are well described, pseudopapillomatous morphology of eyelid SA in an elderly individual is rare and clinically mimics papilloma or carcinoma. This morphological variant and the associated diagnostic dilemma constitute the novelty of our report. The incidence of SA is less than 1% among sebaceous tumors, with the eyelid being an uncommon location [1, 5]. The lesion often presents diagnostic difficulty due to clinical similarity with papillomatous or malignant adnexal lesions.

Our case exhibited a papillomatous external appearance but lacked a fibrovascular core microscopically, instead showing expanded lobules of sebaceous cells beneath stratified squamous epithelium. The basaloid peripheral rim and central mature sebocytes were diagnostic of SA. The absence of mitoses or nuclear atypia differentiated it from sebaceous carcinoma. On clinical examination morphological diagnosis can be made using slit lamp biomicroscopy. Most common morphological features of pathologies are papillomatous, diffuse, nodular with or without ulceration and induration.[6, 7] The association of sebaceous neoplasms with Muir-Torre syndrome has been extensively reviewed in literature, emphasizing the importance of systemic evaluation in affected patients [8, 9]. Shields et al. reported on a large series of sebaceous carcinoma cases, highlighting the clinical challenge in distinguishing benign from malignant sebaceous lesions [10]. Histopathologic evaluation is critical as sebaceous tumors can display a broad spectrum of differentiation, often requiring expertise for accurate classification [11]. A comprehensive understanding of eyelid tumor pathology is essential due to their wide histological variety and potential systemic associations [12]. Sebaceous adenomas may closely mimic sebaceous carcinoma; thus, awareness of these mimics is vital for pathologists [13]. Emerging insights into the pathogenesis of sebaceous tumors suggest roles for molecular alterations in tumor classification and diagnosis [14]. Epidemiological data indicate a rising incidence of sebaceous neoplasms, possibly due to increased recognition and improved diagnostic techniques [15]. In the present case, papilloma was the clinical diagnosis and on histopathology sebaceous gland abnormal proliferation was found. Histopathologically, sebaceous gland proliferation is characterised by vacuolated sebocytes. These are the common abnormalities in the head and neck region which has less propensity for malignant transformation [5]. Pathologies arising from sebaceous gland include hyperplasia, adenoma, carcinoma, sebaceoma. Sebaceous hyperplasia is characterised by papulomatous mature sebaceous gland which are larger than normal. Sebaceous adenoma is a well circumscribed lesion which is located primarily in dermis and sometimes associated with Muir-torre syndrome (MTS). The central sebocytes are more mature than peripheral. The peripheral basal cell layer is thick in SA [6]. SA is differentiated from sebaceomas on the basis of the thickness of the basal cells. If it is more than 50% of the tumor volume then tumors are sebaceomas. Sebaceomas are also well circumscribed lesions located in the dermis with almost no connection with the overlying epidermis. Basal cells are located in the periphery while sebaceous cells in the centre of the lesion and are often associated with MTS but not always like the present case does not have any history of the genetic disease [3, 6]. Often lesions of sebaceous glands are over diagnosed as carcinomatous clinically, hence histopathology becomes an important tool for differentiation [5]. Sebaceous carcinoma on microscopy is an irregular lobular growth composed of atypical sebaceous polygonal cells with a fibrovascular stroma. Central portion of tumor may be necrotic. The well differentiated neoplasm cells have abundant cytoplasm with oval vesicular nuclei with distinct nucleoli while poorly differentiated have high nucleus to cytoplasmic ratio having basaloid growth or infiltrative growth [3].

The spectrum of sebaceous neoplasm is discussed in Comparative Table 1 [6]. Sebaceous hyperplasia, sebaceoma, and sebaceous carcinoma remain key differentials. Sebaceous hyperplasia shows enlarged sebaceous lobules centered on a duct with mature sebocytes and minimal basaloid proliferation. Sebaceoma shows >50% basaloid cells, and sebaceous carcinoma demonstrates cytologic atypia and invasion. IHC analysis further aided in confirming diagnosis. EMA positivity established sebaceous differentiation, while a low Ki67 index (<5%) confirmed low proliferative potential. These findings were consistent with benign behavior and helped to rule out sebaceous carcinoma, which typically exhibits a high Ki-67 index and loss of EMA in undifferentiated regions [16]. Although Muir–Torre syndrome (MTS) is a known association with sebaceous neoplasms, the patient had no family or personal history of visceral malignancies. Routine MMR testing may not be required in elderly isolated cases; however, it is advisable in younger patients, multiple lesions, or unusual histology [7, 9].

**Table 1:** Spectrum of sebaceous neoplasms

Histological features	Sebaceous hyperplasia	Sebaceous adenoma	Sebaceoma	Sebaceous carcinoma
Architecture	Enlarged lobules with central duct	Well-circumscribed lobules	Well-circumscribed, basaloid >50%	Irregular, infiltrative
Sebaceous differentiation	>70%	50–70%	<50%	Variable, often <50%
Basaloid component	Minimal	<50%	>50%	Predominant, pleomorphic
Atypia	Absent	None	Minimal	Marked
Mitoses	Rare	Few	Few–numerous	Numerous, atypical
Necrosis	Absent	Absent	Absent	Common
Invasion	Absent	Absent	Absent	Present (dermal or deep invasion)
Association with No MTS		Possible	Possible	Frequent

## CONCLUSIONS

Sebaceous adenoma of the eyelid is a rare, benign sebaceous neoplasm that can mimic papillomatous or malignant lesions. Its pseudo-papillomatous appearance, low Ki-67 index, and EMA positivity confirm its benign nature. Thorough histopathological and immunohistochemical evaluation is crucial to rule out sebaceous carcinoma and Muir–Torre syndrome. This case adds to literature by describing an unusual papillomatous variant of eyelid sebaceous adenoma in an elderly patient with supportive IHC findings.
